# Autologous full-thickness skin graft as reinforcement in parastomal hernia repair: a feasibility study

**DOI:** 10.1007/s10151-020-02368-6

**Published:** 2020-11-05

**Authors:** V. Holmdahl, U. Gunnarsson, K. Strigård

**Affiliations:** Department of Surgical and Perioperative Sciences, Umeå University, Norrlands Universitetssjukhus, Daniel Naezéns väg, Västerbottens län, 90185 Umeå, Sweden

**Keywords:** Autologous, Full-thickness skin, Parastomal hernia, Hernia repair, IPOM

## Abstract

**Background:**

Parastomal hernia is a common complication of stoma formation and the methods of repair available today are unsatisfactory with high recurrence and complication rates. To improve outcome after surgical repair of parastomal hernia, a surgical method using autologous full-thickness skin grafts as intraperitoneal reinforcement has been developed. The purpose of this study was to evaluate the feasibility of this novel surgical technique in the repair of parastomal hernia.

**Methods:**

A pilot study was conducted between January 2018 and June 2019 on four patients with symptomatic parastomal hernia. They had a laparotomy with suture reduction of the hernia and reinforcement of the abdominal wall with autologous full-thickness skin. They were then monitored for at least 1 year postoperatively for technique-related complications and recurrence.

**Results:**

No major technique-related complications were noted during the follow-up Two patients developed a recurrent parastomal hernia at the long term follow-up. The other two had no recurrence.

**Conclusions:**

Autologous full-thickness skin graft as reinforcement in parastomal hernia repair is feasible and should be evaluated in a larger clinical trial.

## Introduction

Parastomal hernia (PSH) is a common complication after stoma formation, with an incidence in the literature of up to 78% [1–5]. Though only some patients with PSH require surgical repair, the methods available today are unsatisfactory. The use of synthetic mesh as reinforcement has lowered recurrence rates but not complication rates [6]. Recurrence rates as high as 70% are reported in the literature as well as a varying but overall high rate of complications including death [6–8]. The use of synthetic materials can also lead to severe infection in the implanted material, erosion of the intestinal wall and fistula formation, all of which are serious complications associated with significant morbidity [8].

The use of autologous full-thickness skin grafting FTSG) may offer a safer and less expensive alternative, avoiding the problems of foreign material. Autologous full-thickness skin was utilised as reinforcement material in hernia repair during the first half of the twentieth century [9–12] though its popularity waned with the introduction of synthetic mesh material. To our knowledge however, intraperitoneal FTSG as in PSH repair has never been studied. Since the widespread introduction of synthetic mesh interest in the use of FTSG in hernia repair has faded, and the technique has mainly been used in last resort cases [13, 14]. However, considering the short- and long-term complications related to conventional synthetic mesh in PSH repair, it is time to take a critical review of existing techniques and to consider better alternatives.

Prior to testing FTSG as reinforcement in humans, our research group carried out preclinical studies to ensure the safety of the method. A mouse model showed good FTSG survival in both onlay and intraperitoneal positions in the abdominal wall [15]. We also conducted experiments on fresh skin samples showing that skin has remarkable tensile strength—an important property for reinforcement material in hernia surgery [16]. Histological studies from the 1930s showed no signs of malignant transformation or cyst formation related to burying full-thickness skin in humans [17, 18].

This pilot study on four humans is the first to test the feasibility of using FTSG as intraperitoneal reinforcement in the repair of PSH.

## Materials and methods

### Patients

Patients with suspected PSH on the waiting list for assessment by a specialist in abdominal wall surgery at our university hospital were considered for inclusion. Inclusion criteria were symptomatic end colo-, ileo- or urostomal hernia and age above 18 years. Exclusion criteria were: cognitive difficulties making it difficult to follow postoperative instructions; limited access to good quality skin; expected high donor site morbidity; parastomal fistula; or Crohn’s disease. Four patients were recruited.

### Operative technique

All operative procedures where preformed under general anaesthesia. Epidural analgesia was used as a complement and continued postoperatively. Our intention was to treat all patients with trimethoprim-sulphonamide 800 mg/160 mg as antibiotic prophylaxis, but this was replaced by cefotaxime 1000 mg in patients 2 and 3 due to a history of acute intermittent porphyria and allergy to sulphonamide respectively. Furthermore, all patients received metronidazole 1200 mg preoperatively.

**Fig. 1:**
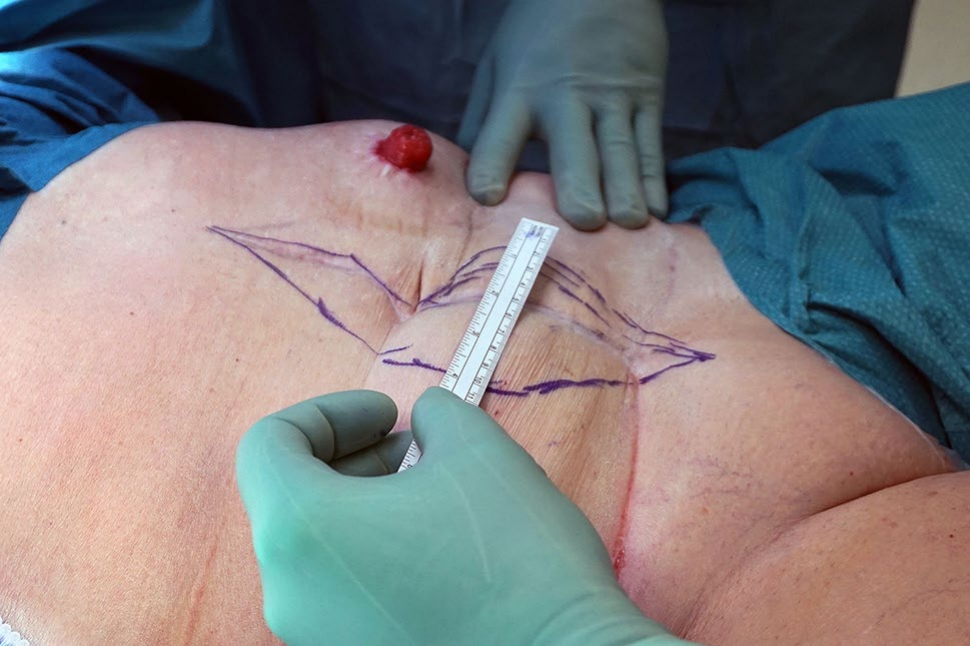
Area of skin intended to be transplanted. Old midline scar included but umbilicus excluded

**Fig. 2:**
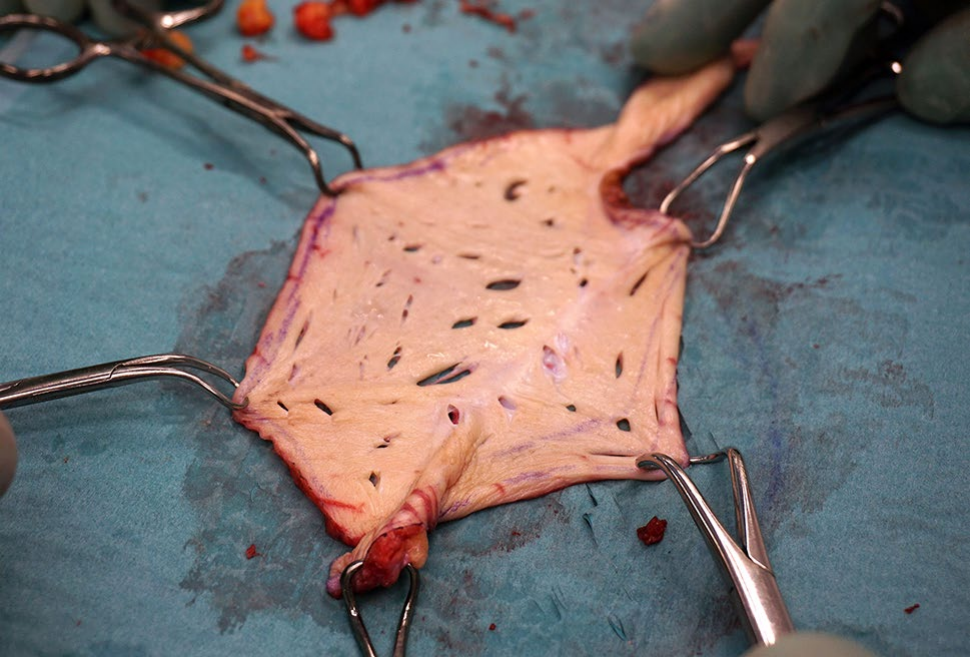
Skin graft after knife-meshing, 13 × 10 cm

Surgery began with marking of the area of skin intended to be harvested for the FTSG, preferably above the midline (Fig. 1). To be able to obtain adequate overlap the width of the graft had to be at least 8 cm. After harvesting, the skin graft was then prepared by sharp dissection of all macroscopically visible subcutaneous tissue leaving the dermis and epidermis intact. Multiple small incisions, 0.5–1 cm in length, were made in the graft (Fig. 2) to prevent seroma and hematoma formation and to increase the area of the graft [19, 20]. A larger incision related to the size of the stomal intestine was made at the centre of the graft. The graft was then placed in surgical gauze soaked in hydrogen peroxide until final application.

A midline laparotomy was performed to ensure adequate access for the procedure. The stoma was then dissected from the skin and sealed with a temporary running suture. Complete adhesiolysis was performed around the stoma and the stoma was retracted into the abdominal cavity. The stoma fascial defect was reduced to approximately 25–30 mm, depending on the type of stoma, with interrupted single.

**Fig. 3:**
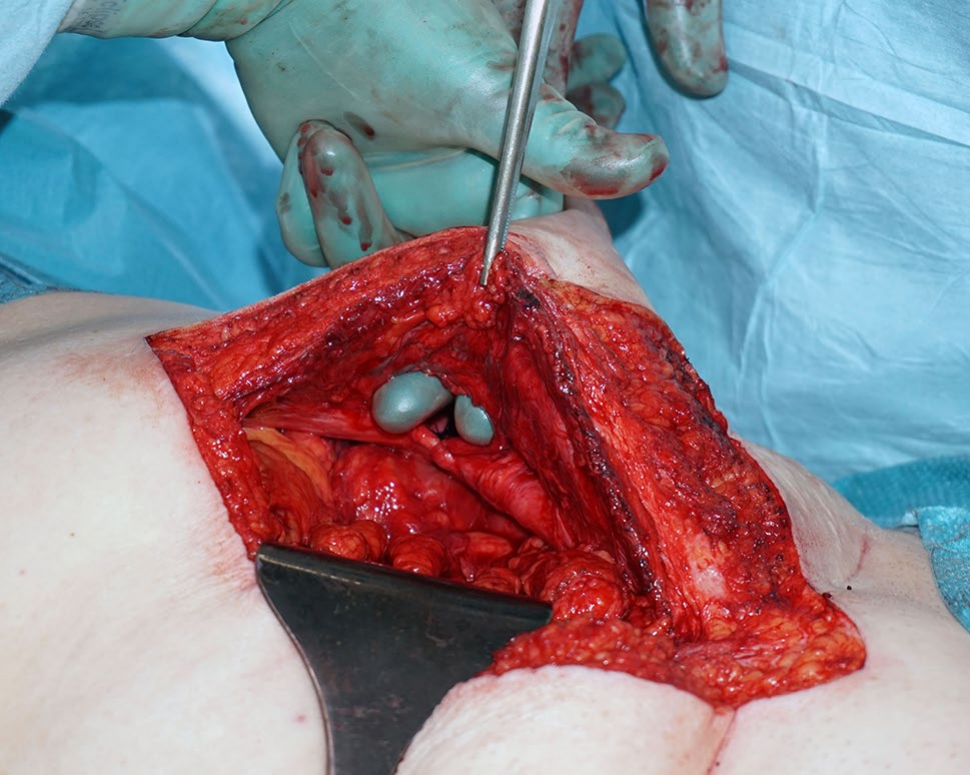
The fascial defect on the patients left side after reduction, seen from the midline laparotomy. Note the suture line with interrupted 2-0 PDS sutures seen laterally in the defect

**Fig. 4:**
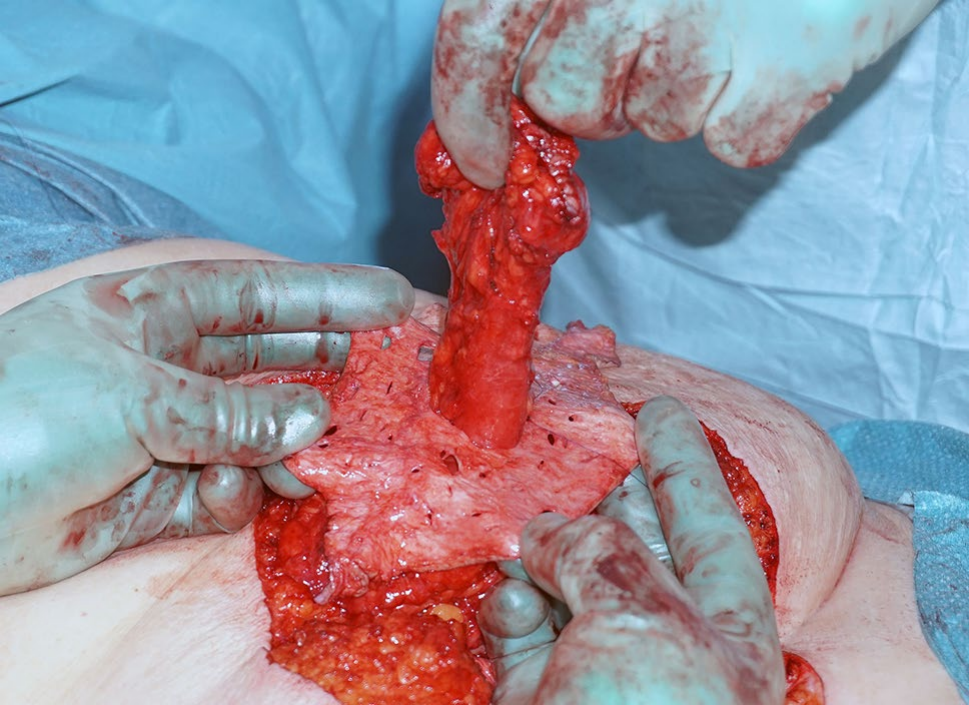
Skin graft threaded onto the stomal intestine at the distance measured from the fascia to the skin surface

2-0 *PDS* (Ethicon, Cornelia, GA, USA) sutures (Fig. 3). The FTSG was then threaded over the stomal intestine at the distance measured from the fascia to the skin surface plus additional margin to enable stoma maturation, and anchored in place with interrupted single 3-0 monocryl (Ethicon, Cornelia, GA, USA) sutures (Fig. 4).

**Fig. 5:**
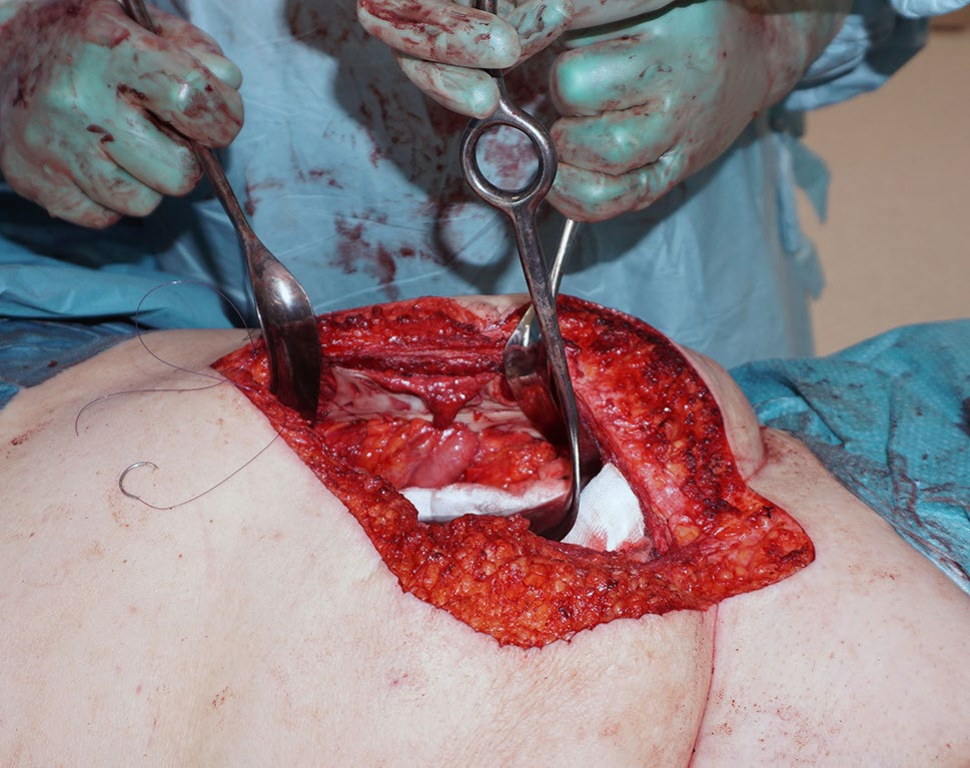
Skin graft on place

The stomal intestine was drawn back through the fascial defect and the FTSG sutured to the abdominal wall with interrupted 2-0 polydioxanone sutures along the edges of the graft at an interval of 10–20 mm (Fig. 5) making sure the graft was adapted under tension. The importance of adapting the skin graft under tension has been emphasized by previous authors since their clinical experience was that the tension reduced the risk of dermoid cysts and seroma formation [11, 22]. Our aim was to obtain a 5 cm overlap around the reduced fascial defect. According to the latest guidelines from the International Endohernia Society, the mesh area-to-defect area ratio in ventral and incisional hernia repair is of major importance to reduce the risk of recurrence [23]. In the case of parastomal hernia repair, however, where a permanent defect must be left for the stoma bowel, these guidelines are not applicable. Repair of the fascial defect was reinforced with sutures running through both the FTSG and both sides of the fascial defect. A running 2-0 polydioxanone suture was used to close the fascia in the midline laparotomy incision.

### Postoperative management

All 4 patients were given subcutaneous 4500 IE *tinzaparin* daily as thrombosis prophylaxis. Patients one, two and four received prophylaxis for 10 days after discharge from the hospital. Patient three, however, had suffered chronic pain for several years prior to surgery causing a longer period of immobilisation, and was thus treated for 24 days.

### Follow-up

The first follow-up was performed by telephone 2 weeks after surgery. Clinical follow-up was conducted at least 1 month after surgery and around 1 year after the procedure. Noted outcomes included the occurrence of any surgical complication (such as infection, seroma/hematoma, stoma complication, reoperation), pain, satisfaction, aesthetic outcome and stomal recurrence. Patients were also offered the opportunity of a follow-up after 36 months for long-term outcome data. Since this study was of an experimental nature and the aim was to discover procedure-related complications, patients could be called back for extra follow-up visits at any sign of complication suspected by the patient or the ostomy nurse. Thus, a patient may have had more follow-up visits than stated in the protocol. Patients were examined for any sign of complication or recurrence. At the 1-year follow-up, a stomal ultrasound was performed to rule out the presence of recurrence. Stomal ultrasound has been shown to have high sensitivity and specificity for parastomal hernia and was primarily performed to avoid exposing the patient to ionising radiation. If the results of the ultrasound were inconclusive, a computed tomography (CT) scan was also performed [24].

### Statistics

All patient characteristics, perioperative information and follow-up information were collected in an Access database (Microsoft, Redmond, WA, USA).

## Results

Four patients were included in this pilot study from January 2018 to June 2019. Baseline characteristics are shown in Table 1.

**Table 1:** Baseline characteristics

Pilot patient no	1	2	3	4
Age, years	82	72	60	65
Sex	Female	Male	Female	Male
BMI, kg/m^2^	30,1	32,8	29	24,9
Stoma	End sigmoid colostomy	End sigmoid colostomy	End ileostomy	End sigmoid colostomy
Reason for stoma	Perforated Diverticulitis, Hartmann procedure	Rectal cancer, APR	Treatment refractory obstipation, total colectomy	Rectal cancer, APR

*BMI* Body mass index, *APR* Abdominoperineal resection

The size of the hernia varied considerably (50,3–12,6 cm^2^) but the area of the skin graft was more uniform (94,2–106,8 cm^2^) (Table 2). In no case was there difficulty in adapting the skin wound after harvesting of the FTSG.

**Table 2:** Interventional data

Pilot patient no	1	2	3	4
Intraop. Area of hernia (cm^2^)	50,3	19,6	12,6	12,6
Area of FTSG (cm^2^)*	102,1	100,5	94,2	106,8
Length of bowel above FTSG (cm)	7	8	12	9
Operating time (minutes)	286	267^†^	204	163
Length of hospital stay (days)	3	3	4	4

*FTSG* Full-thickness skin graft

*Considered an ellipse

^†^Operation was prolonged 30 min due to deficient adherence to operation theatre routines

Operating time decreased by 123 min from the first case to the last. Surgical time for Patient two was prolonged 30 min due to a swab being left in the abdomen after skin closure. Patient four had a small gap between the stoma and the skin which was noted prior to discharge. The gap was corrected on the ward with a suture under local anaesthesia (Table 3).

**Table 3:** Clinical follow-up

Pilot patient no	1	2	3	4
Recurrence	Asymptomatic	Symptomatic	No	No
Length of follow-up (months)	25	23	12	12
Surgical complication*	No	No	No	Yes

*Infection, seroma/haematoma, stoma complication, reoperation

Patient one noted a bulge adjacent to the stoma 18 months after surgery. At clinical follow-up 25 months after surgery, a small asymptomatic recurrence was seen on stomal ultrasound. Patient two noticed a small asymptomatic bulge 10 months after surgery. Eleven months later the patient returned to the hospital with small bowel obstruction due to a herniated loop of small bowel that was manually reduced in the emergency room causing immediate relief of symptoms. After a thorough medical history, it was discovered that the patient had suffered a period of profuse vomiting due to an attack of acute intermittent porphyria less than a week after surgery. During this episode, the patient felt that something gave way next to the stoma.

Patients three and four have well-functioning stomas with no sign of recurrence at the latest follow-up.

## Discussion

This pilot study presents four patients in whom autologous FTSG was used as intraperitoneal reinforcement in a novel surgical technique for the repair of PSH. The lack of major method-related complications suggests the method is feasible in humans.

Patient two returned with symptomatic recurrence probably caused by excessive vomiting in the early postoperative period. The main aim of PSH repair is to relieve the patient of symptoms, and it is therefore most important to balance the potential benefits of the procedure against the risks associated with surgery. Recurrence is seen as failure of a repair, but it can be argued that it is not a complication. Furthermore, recurrence per se does not automatically imply a decrease in quality of life if it is asymptomatic, as in Patient one.

Since the study included four patients without a control group, it is not possible to draw any reliable conclusions on the frequency of recurrence. Considering the results of currently used methods of repair, recurrences are so frequent as to be expected even in small case series such as this.

Patient four required minor revision of the stoma that was performed on the ward, and this event was not necessarily related to the method used. Due to the size and nature of this study, no effort was made to quantify the method’s effect on quality-of-life, which is a major outcome when evaluating a novel method in a larger setting. The most important finding of this feasibility pilot study is the absence of major procedure-related complications, which is a considerable drawback of the methods currently used [8].

Despite its popularity in the early twentieth century, there are few reports of skin grafting in hernia surgery today, though there are few studies comparing outcomes of hernia repairs using synthetic mesh or FTSG. Meshed split-thickness skin grafts are often used in cases where skin coverage of a wound is required such as in burns, after extended excision of a skin malignancy or other wound repair [19, 20]. In these cases, the main purpose is to replace damaged or absent skin, not to obtain tensile strength as in hernia repair. When harvesting split-thickness skin grafts only the epidermis and a part of the dermis is excised enabling the skin to heal as a secondary wound, reverting to its original configuration. This provides an abundant source of grafting material. FTSG, on the other hand, requires complete excision and primary closure which limits the amount of grafting material available. However, in most patients there is enough abdominal skin to allow for excision of a graft large enough for the hernia repair. In the four pilot cases in this study, there were no problems with skin closure after excision of the FTSG. This was also the case in the 24 patients included in an randomized controlled trial comparing FTSG to synthetic mesh in giant incisional hernia repair [25].

The purpose of reinforcement material in hernia surgery is to increase mechanical strength and to add fibrous tissue to the weakened fascial structures. Most of the mechanical strength of skin is derived from the dermis. FTSG is therefore preferred to split-thickness skin graft to retain as much mechanical strength as possible.

The technique used in the present study included sutured reduction of the fascial defect, which we believe is important because it provides direct contact between the entire skin graft and the richly vascularised peritoneum, encouraging rapid ingrowth and metabolic support of the biological graft. There is also reason to believe that meshing of the graft could increase the rate of vascularisation and thereby improve graft adaptation [21]. Defect reduction also decreases the area the skin graft must cover with adequate overlap around the repair. Since defect reduction is not a part of primary repair of the PSH, we chose a slowly resorbable suture rather than a non-resorbable suture to avoid long-lasting foreign material in the abdominal wall.

A possible disadvantage of FTSG is that it is transformed after implantation, and little is known about how metamorphosis affects its physical properties. From histological investigations we know that the skin transforms into fibrous tissue after implantation [18, 26] but we do not know how mechanical properties of the graft change with time. Should autologous FTSG heal rather than scar in situ this would lead to better tissue integration into the abdominal wall. Such tissue remodelling and integration is highly desirable in PSH repair to create a continuum between the stomal bowel and the abdominal wall, thereby preventing recurrence between the reinforcing implant and the stoma bowel.

The definitive safety of a novel surgical method cannot be determined from a small pilot trial such as this. Intra-abdominal surgery is always associated with risk for serious complications that are rare but can be fatal. The aim of this study was to introduce the concept of intra-abdominally placed autologous FTSG, to ensure that it is technically possible to perform, and that the method has no major method-related complications. Animal studies and in vitro mechanical experiments preceded this pilot application in humans; a link in a chain of studies aiming to evaluate the use of FTSG in parastomal repair and to compare the method with those currently available [15, 16].Considering the lack of major method-related complications we conclude that this FTSG method is feasible. The results of the study along with previous preclinical experiments provide enough evidence to proceed with this novel method in a larger clinical trial. The forthcoming randomised controlled trial named SHIFT (Stoma Hernia Intraperitoneal Full-Thickness skin grafting), is registered at clinicaltrials.gov (ID NCT03667287).

## Data Availability

Not applicable.
